# Pomegranate and Cherry Leaf Extracts as Stabilizers of Magnetic Hydroxyapatite Nanocarriers for Nucleic Acid Delivery

**DOI:** 10.3390/ijms262311562

**Published:** 2025-11-28

**Authors:** Hina Inam, Simone Sprio, Federico Pupilli, Marta Tavoni, Anna Tampieri

**Affiliations:** Institute of Science, Technology and Sustainability for Ceramics, National Research Council, 48018 Faenza, Italy; hina.inam@issmc.cnr.it (H.I.); federico.pupilli@issmc.cnr.it (F.P.); marta.tavoni@issmc.cnr.it (M.T.)

**Keywords:** magnetic hydroxyapatite, iron doping, nucleic acids carrier, nanomedicine, leaf extracts, colloidal stability

## Abstract

Small interfering RNAs (siRNAs) provide strong therapeutic potential due to their efficient gene-silencing properties; however, their instability limits clinical application. Nanoparticle carriers may overcome this problem; in particular, magnetic nanoparticles show great promise as they can be directed to the target sites by external magnetic fields, thus improving delivery efficiency and reducing off-target effects. In addition, magnetic nanoparticles offer a novel nanoplatform for theranostic applications, integrating siRNA delivery with magnetic resonance imaging and magnetic hyperthermia for synergistic diagnostic and therapeutic advantages. The present work reports the development of a novel platform based on biomimetic magnetic nanoparticles made of Fe(II)/Fe(III)-doped apatite (FeHA) nucleated and grown in the presence of cherry and pomegranate leaf extracts to enhance the colloidal stability and make it suitable for nucleic acid delivery under the guidance of magnetic fields. This approach allowed the obtention of FeHA suspension with increased negative zeta potential leading to very good stability. In addition, the functionalization with natural extracts conferred antioxidant properties also favoring the maintenance of the Fe(III)/Fe(II) ratio in the apatitic structure, inducing the superparamagnetic properties. To evaluate the delivery capability of the system, a model GAPDH-targeting siRNA molecule was employed. Its interaction with the nanoplatform was characterized by assessing loading capacity and release kinetics, which were further interpreted using mathematical modeling to elucidate the underlying release mechanisms.

## 1. Introduction

There is a persistent interest in using small interfering RNAs (siRNAs) as a therapeutic approach because of their ability to selectively silence disease-causing genes with high specificity and efficacy. siRNAs silence sequence-specific genes through RNA interference, a naturally occurring biological mechanism in cells that inhibits gene expression at the post-transcriptional stage [1]. The therapeutic efficacy of siRNAs has been shown in the treatment of several disorders, including tumors and infections [2]. The main benefits of siRNAs over conventional small therapeutic molecules are their high potency and ability to target “non-druggable” targets, such as proteins without an enzymatic function or with an unreachable conformation, compared to conventional drug molecules [3]. Furthermore, siRNAs can be tailored to affect almost any target gene. The synthesis of siRNAs is relatively straightforward and does not require a cellular expression system, complicated protein purification, or re-folding processes, as compared to other types of RNA therapeutics, such as antibodies or protein-based drugs. RNA-based gene therapy has made significant advancements over the last decade, resulting in the authorization of siRNA-based therapeutic compounds [4].

The clinical translation of siRNA-based therapeutics faces significant challenges, particularly in delivery and manufacturing [5,6]. Effective therapeutic outcomes require the successful delivery of siRNA to the target site at an optimal concentration, which is challenging because of the peculiarities of siRNA. Key challenges include rapid degradation by nucleases, short lifetime in the bloodstream, limited cellular uptake due to restricted membrane permeability, off-target gene silencing, rapid renal clearance, potential stimulation of the immune system, and emergence of escaping mutant viruses [6]. The development of safe and efficient methods to deliver small interfering RNAs to their target cells has been a focus of research in recent years.

Biomaterials and nanoparticles (NPs) have been successfully designed and optimized for siRNA delivery [7]. NPs can be designed to prevent the in vivo enzymatic degradation of siRNAs, improve cellular uptake, facilitate targeted delivery, extend circulation time, enhance stability, promote accumulation in tumor tissue, and enable controlled release. Among these, hydroxyapatite (HA)-based NPs have gained attention as a suitable siRNA carrier because of their excellent biocompatibility, easy internalization into cells, and ability to gradually dissolve under physiological or slightly acidic conditions, allowing controlled release of the payload. Moreover, HA surface chemical composition enables efficient siRNA loading and protection during circulation [8,9]. HA functional characteristics can also be readily tailored either by introducing capping agents during HA NP synthesis or by applying post-synthetic functionalization techniques, both of which aim to enhance colloidal stability and improve NP circulation at the delivery site. At this regard, citrate-stabilized HA NPs (Cit-HA NPs) are extensively used due to the strong negative surface charge, which inhibits particle aggregation and maintains their dispersion in water. The negative surface charge facilitates the modification of the particle surface via electrostatic interactions, enabling Cit-HA NPs to effectively deliver negatively charged molecules such as nucleic acids and other therapeutic agents [10]. Polyethylene glycol (PEG) has been used to coat HA NPs, enhancing their stability and improving their circulation duration in the bloodstream, whereas chitosan, a positively charged biopolymer, facilitates binding to negatively charged molecules such as nucleic acids via electrostatic interactions, thus promoting cellular uptake [11,12]. There is a growing interest in developing surface modifiers that are both environmentally friendly and biologically active, as conventional methods of functionalization depend on synthetic reagents and have limited inherent bioactivity.

An intriguing application of NP delivery is the use of magnetic NPs, particularly superparamagnetic iron oxide NPs (SPIONs), for magnetic guidance, facilitating non-invasive, site-specific targeting through external magnetic fields and providing theragnostic potential through magnetic resonance imaging [13]. Although SPIONs show potential in improving siRNA transfection efficiency, their clinical use is impeded by the need of extensive surface functionalization to achieve biocompatibility, as well as concerns about long-term toxicity due to organ accumulation, particularly in the liver and kidneys [14,15].

In this scenario we have developed iron-doped hydroxyapatite nanoparticles (FeHA NPs) featuring high biocompatibility, bioactivity, and superparamagnetic properties, given by co-substitution with Fe^3+^ and Fe^2+^ ions in the sites of calcium in the apatite lattice, raising the superparamagnetic properties [16]. To enhance colloidal stability, FeHA NPs were synthesized in the presence of biomolecules extracted from pomegranate and cherry leaves, selected as reducing, stabilizing, and capping agents. Cherry leaf extract is used in the cosmetic, food, and pharmaceutical industries due to its rich content of bioactive compounds like phenolic acids, flavonoids, and tannins. These compounds confer anti-inflammatory, antioxidant, and astringent properties, making it useful for skin and hair health, managing blood glucose levels, and potentially treating wounds and preventing bacterial growth. On the other hand, pomegranate leaf extract contains beneficial phytochemicals, such as phenols, flavonoids, tannins, and alkaloids, which exhibit antioxidant, anti-inflammatory, antimicrobial, and anticoagulant properties. These properties suggest potential applications in the pharmaceutical, cosmetic, and nutraceutical industries for treating various health conditions like cardiovascular disease, diabetes, and skin [17]. Thanks to their properties, the use of biomolecules derived from plants can be considered as a green and cost-effective approach for various therapeutic purposes. In this work, we investigated these bioactive extracts as functionalizing agents for FeHA NPs to improve their colloidal stability and capability to load and release, with sustained time profiles, a model positive control siRNA. GAPDH-targeting siRNA was employed as a neutral benchmark to enable predictable, non-therapeutic evaluation and optimization of nanocarrier performance. The aim of this study is a preliminary assessment of the physicochemical and colloidal properties of FeHA NPs and the ability of the nanoplatform for controlled siRNA loading, protection, and release.

## 2. Results and Discussion

### 2.1. Characterization of Plant Extract

**Figure 1:**
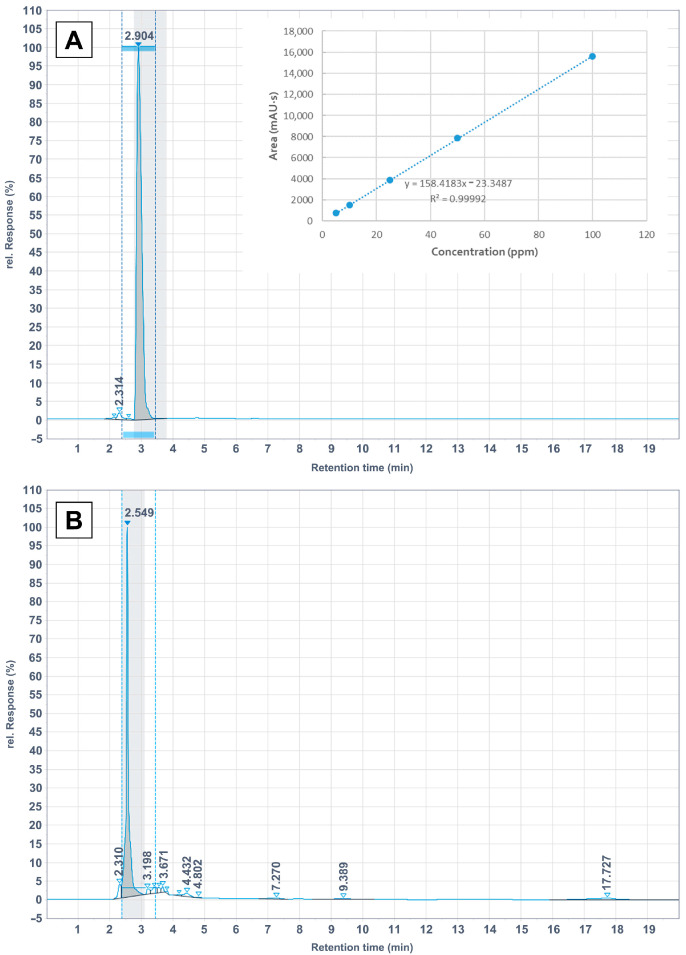
Chromatogram for gallic acid reference standard at 100 ppm concentration with the corresponding calibration curve (**A**) and chromatogram for pomegranate leaf extract at 500 ppm concentration (**B**).

In this study, a quantitative analytical approach was preliminarily assessed to investigate the molecular constituents of the pomegranate leaf extracts using an HPLC–UV system. Based on the literature, gallic acid was expected to be one of the predominant phenolic compounds, together with tannins, anthocyanins, flavonoids, and terpenes [18,19]. The calibration curve was developed to quantify the gallic acid in the plant extracts (reported in the upper right corner of Figure 1A. The established method exhibited good linearity, reproducibility, and sensitivity within the tested concentration range.

However, the HPLC–UV chromatograms of the crude extracts revealed a major peak eluting at approximately 2.5 min, which differed slightly from the retention time of the gallic acid standard (2.9 min) (Figure 1A,B). This deviation suggests that the pomegranate leaf extracts comprise a complex mixture of phenolic and flavonoid derivatives, potentially glycosylated species, which may alter their chromatographic behavior under the employed neutral pH extraction conditions. Cherry leaf extract is known to contain a complex mixture of secondary metabolites, including alkaloids, flavonoids, saponins, tannins, and terpenoids. [20,21]. Comparable chromatographic profiles have been reported for *Punica granatum* and *Prunus cerasus* leaves, where broad polyphenolic fingerprints were observed rather than the dominance of a single compound [22,23,24].

Since the primary objective of this work was to evaluate the functional capability of pomegranate and cherry leaf extracts as biogenic redox and capping agents in the synthesis of FeHA NPs, an exhaustive phytochemical profiling was beyond the intended scope. Instead, extract characterization was complemented by UV–Vis spectroscopy, which confirmed the presence of characteristic absorption bands associated with phenolic and flavonoid chromophores (around 520 nm), and by the DPPH radical scavenging assay, which provided a quantitative measure of the total antioxidant potential. These analyses sufficiently demonstrate the extracts’ electron-donating and stabilizing capabilities, which are key physicochemical properties underpinning their effectiveness in the green synthesis process.

While complete identification of the individual phytoconstituents would provide further insight, it should be noted that similar studies on plant-mediated NP synthesis commonly rely on UV–Vis and antioxidant assays as reliable indicators of reducing strength [25,26,27,28]. Therefore, the combination of UV–Vis spectroscopy, DPPH analysis, and qualitative HPLC–UV profiling may be considered sufficient to provide evidence of the presence of active phytochemicals involved in the biogenic synthesis of FeHA NPs.

The antioxidant potentials of the pomegranate and cherry leaf extracts were determined with the DPPH radical scavenging assay. The DPPH technique is straightforward and based only on the interaction between the DPPH radical and the antioxidant [29].

**Figure 2:**
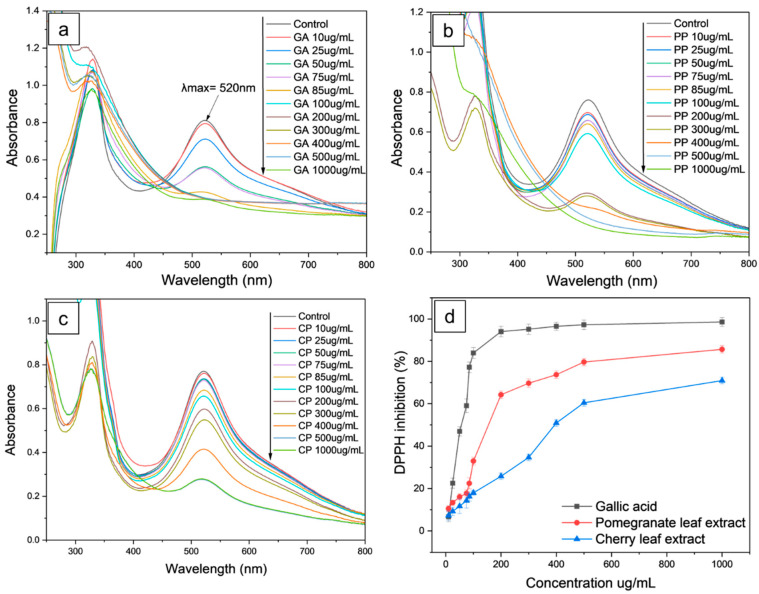
Absorption spectra of gallic acid standard (**a**), spectra of pomegranate and cherry leaf extract (**b**,**c**), and antioxidant concentration-dependent DPPH inhibition (**d**).

Figure 2a–c show the DPPH radical scavenging potential of the gallic acid standard and both plant leaf extracts at different concentrations. All samples exhibited antioxidant properties, with scavenging activity increasing in a concentration-dependent manner. Upon reduction by antioxidants, the DPPH solution turns from purple to yellow, indicating that free radicals have been scavenged. Additionally, the decrease in absorbance at 520 nm confirms this reduction.

IC50 is defined as the concentration of extracts exhibiting antioxidant activity able to inhibit free radicals (DPPH) by 50% and is determined by fitting a sigmoidal curve to the plot of DPPH scavenging percentage versus the extract concentration. Lower IC50 values indicate superior antioxidant activity of the extract [30]. It is well-known that phenolic contents, particularly flavonoid components, impact the antioxidant activity of plant extracts [31,32].

In this work, gallic acid was used as a standard antioxidant to evaluate the radical scavenging efficiency of the plant extracts, showing the higher potency in neutralizing free radicals with an IC50 value of 54.23 µg/mL (Table 1). In comparison, the pomegranate leaf extract showed moderate antioxidant potential with an IC50 of 145.84 µg/mL, while the cherry leaf extract had the lowest antioxidant potential, as evidenced by the highest IC50 value of 349.3 µg/mL.

These results suggest that the higher PLE antioxidant capacity compared to CLE could likely be attributed to its higher concentration of phytochemicals, particularly phenolic compounds [33]. Such compounds are known for their strong radical scavenging activity due to their resonance-stabilized structure, phenol moiety, and capacity to donate an electron or hydrogen atom to free radicals [34].

### 2.2. Characterization of Pure Hydroxyapatite, FeHA, CP-FeHA, and PP-FeHA

FeHA NPs were functionalized with the above characterized phytochemicals extracted from cherry and pomegranate leaves.

**Figure 3:**
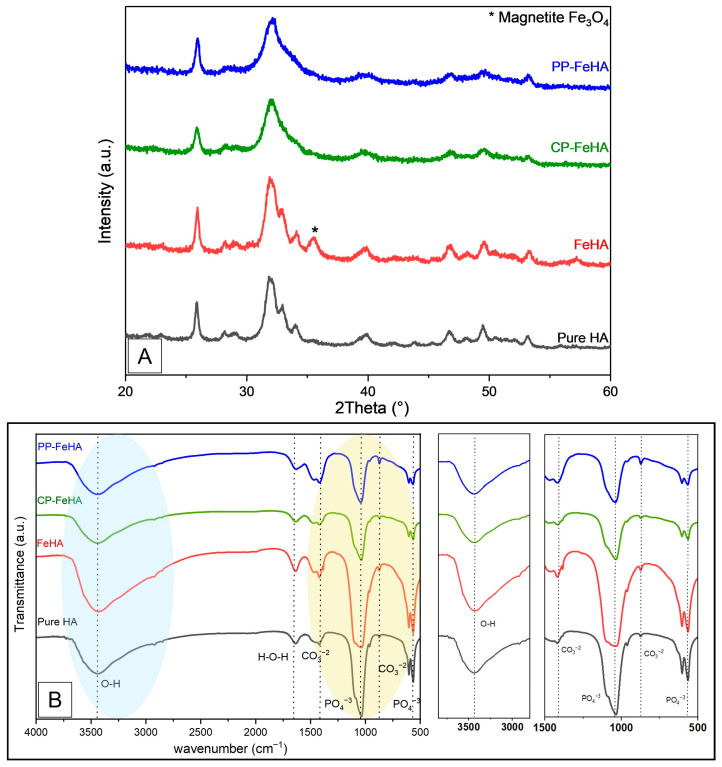
(**A**) PXRD patterns and (**B**) FTIR spectra of pure HA, FeHA, CP-FeHA, and PP-FeHA.

PXRD analysis (Figure 3A) of FeHA, CP-FeHA, and PP-FeHA in comparison to control HA confirms that apatite is the main crystalline phase in all samples. Relative to HA, all Fe-substituted samples exhibited broader diffraction peaks, particularly pronounced in those synthesized using plant extracts, likely indicating a reduction in crystallinity. This trend is further supported by the splitting factor values derived from FTIR analysis as previously described [35], which provide an estimate of the overall apatite crystallinity index, showing a gradual decrease from 3.43 for HA to 3.04 for FeHA, and 2.45 for PP-FeHA and 2.26 for CP-FeHA.

Upon doping with iron, FeHA shows slightly broadened yet distinct hydroxyapatite peaks, indicating moderate crystallinity and successful iron incorporation without major structural disruption. Furthermore, the FeHA sample features traces of a secondary phase, assignable to iron oxides such as magnetite or maghemite phases, as previously reported in the literature [16]. The lower crystallinity is likely due to lattice strain and reduced crystallite size caused by partial replacement of Ca^2+^ with Fe ions within the HA lattice. FeHA synthesized in the presence of plant extracts (CP-FeHA and PP-FeHA) exhibits more pronounced peak broadening and further reduced peak intensity, suggesting a decrease in the FeHA crystal ordering ascribed to physicochemical interactions between plant extracts and reactants during the synthesis process, affecting the nucleation and growth of the apatitic phase. Bioactive compounds such as polyphenols and flavonoids present in the extracts may chelate metal ions, reducing iron oxides formation, and interfere with orderly crystal growth, leading to smaller crystallites and increased lattice strain [36].

Structural characterization was also performed by FTIR spectroscopy (Figure 3B), which presents the spectra of pure HA, FeHA, CP-FeHA, and PP-FeHA in the range of 4000–500 cm^−1^, highlighting key functional groups and compositional variations resulting from Fe incorporation and plant-derived modifications. All samples exhibited the characteristic vibrational band of apatite, i.e., the main mode ν_3_PO_4_ as a broad band centered at ca. 1049 cm^−1^, as well as the double-split band at 598 and 565 cm^−1^ of ν_4_PO_4_ mode, confirming structural integrity, while slight intensity differences in CP-FeHA and PP-FeHA, also indicated in XRD, suggest surface modifications affecting lattice disorder or crystallinity. Additional absorption bands observed at 879 and 1400–1500 cm^−1^ correspond to the v_2_CO_3_ and ν_3_CO_3_ vibrations of carbonate ions in the apatitic lattice (Type B carbonate substitution due to the dissolution of CO_2_ in solvent during precipitation). All samples showed a broad absorption band at 3400–3550 cm^−1^, ascribed to O-H stretching vibrations of structural hydroxyl groups and adsorbed water. In comparison to HA, the intensity of this band decreased gradually in FeHA, CP-FeHA, and PP-FeHA, suggesting either possible substitution of OH^−^ groups or changes in hydrogen bonding interactions, most likely resulting from surface modifications induced by the leaf extracts and by iron substitution. Additionally, a band at 1633 cm^−1^ is attributed to the νH_2_O bending mode of adsorbed water on the HA surface.

**Figure 4:**
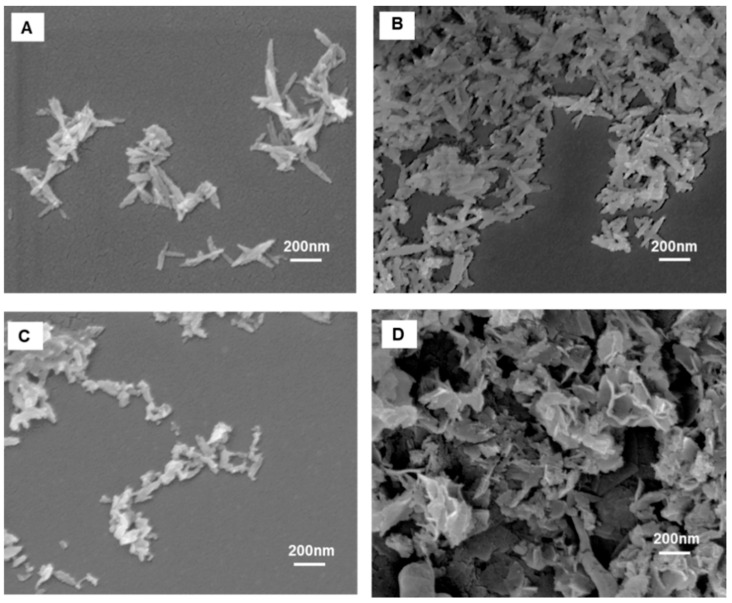
FE-SEM micrographs of (**A**) HA, (**B**) FeHA, (**C**) CP-FeHA, and (**D**) PP-FeHA.

Morphology of the materials was observed by FE-SEM (Figure 4). The micrographs reveal that HA NPs exhibit elongated, needle-like morphology. Upon iron incorporation (in FeHA sample), the particles become noticeably shorter in length while maintaining a similar width. The introduction of cherry leaf extract results in even more compact and less elongated structures, indicating a progressive reduction in particle aspect ratio. CP-FeHA, synthesized from cherry leaf extract with moderate levels of natural antioxidants as assessed by colorimetric assay, maintained a plate-like morphology with improved dispersion, most likely due to the stabilizing phytochemicals, like flavonoids and gallic acid. In contrast, PP-FeHA, synthesized using pomegranate leaf extract with a high natural antioxidant amount, exhibited aggregated clusters and evident morphological changes, indicating a significant effect of plant-derived polyphenols on nanostructure formation and surface functionalization.

Chemical composition of the synthesized materials is shown in Table 2. The FeHA materials exhibit the presence of iron in both Fe(III) and Fe(II) oxidation states, as determined by ICP-OES and UV–Vis spectroscopy. Further confirmation of Fe doping is shown by the reduction in the Ca/P molar ratio relative to stoichiometric hydroxyapatite, likely due to Fe-to-Ca substitution. Moreover, CP-FeHA and PP-FeHA showed intermediate Ca/P ratios and elevated (Ca + Fe)/P ratios compared to undoped HA, confirming successful incorporation of iron into the hydroxyapatite structure. The iron content varied across the samples, with FeHA showing the highest overall Fe content, followed by PP-FeHA and CP-FeHA, where the presence of the leaf extracts seemed to inhibit Fe incorporation. Notably, both plant extract-stabilized samples exhibited higher Fe(II) fractions than FeHA, suggesting that the reducing environment provided by natural antioxidants likely led to Fe(II) stabilization. Among these, PP-FeHA displayed the highest Fe(II) content, which can be attributed to the greater antioxidant capacity of the pomegranate leaf extract, as also reflected by its relatively lower IC50 (in Table 1). The enhanced reducing conditions during synthesis thus likely favored the retention of Fe(II), resulting in its elevated relative content.

Colloidal stability of the samples was assessed by DLS and electrophoretic mobility measurement (Table 3). DLS analysis revealed a narrow, monomodal particle distribution for all prepared samples (pH ~7). The notable reduction in particle size and PDI, coupled with the enhanced surface charge, particularly in CP-FeHA and PP-FeHA, represents improved colloidal stability, prevents agglomeration, and facilitates efficient dispersion in solutions, due to the bioactive compounds present in the plant extracts.

The specific surface area of the materials (SSABET) determined by nitrogen gas adsorption is reported in Table 2. Hydroxyapatite showed a surface area of 97.18 m^2^/g, which slightly increased to 102.65 m^2^/g upon iron doping (FeHA). However, following stabilization with natural stabilizers, the surface areas of CP-FeHA and PP-FeHA substantially increased to 182.00 m^2^/g and 235.75 m^2^/g, respectively, plausibly attributed to the effect of natural biomolecules present in the plant extracts, able to decrease particle–particle aggregation and hence increase the specific surface area.

**Figure 5:**
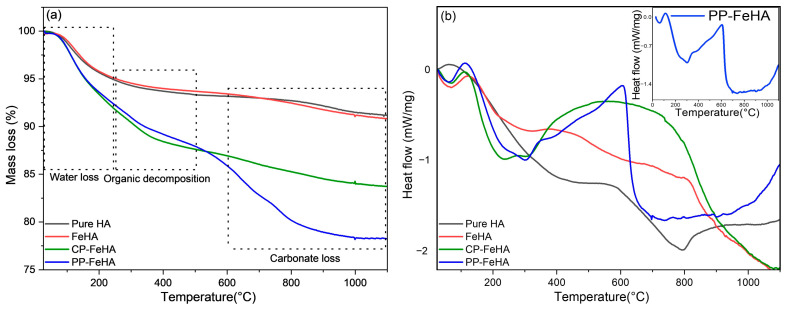
TGA (**a**) and DSC analysis (**b**) of the obtained samples.

The content of volatile species was evaluated by TGA (Figure 5 and Table 3). HA and FeHA exhibit two mass losses: one occurs between room temperature and 250 °C, associated with the removal of adsorbed and structural water, and another between 600 and 1100 °C, corresponding to the decomposition of carbonate ions inside the structure [37]. On the other hand, CP-FeHA and PP-FeHA show more prominent mass losses at lower temperatures as well as an additional weight loss between 250 and 500 °C, which was attributed to organic decomposition, likely due to the presence of plant-derived organic compounds [38], which was quantified as ca. 4.9 and 6.4 wt.%, respectively. In comparison to HA and FeHA, the higher carbonate loss in CP-FeHA and PP-FeHA indicates CO_3_^2−^ incorporation from plant-derived organic acids during synthesis, which substitutes phosphate or hydroxyl groups in the hydroxyapatite lattice and decomposes at high temperatures, thus contributing to increased mass loss. DSC curves supported these findings, exhibiting exothermic peaks between 250 and 600 °C for CP-FeHA and PP-FeHA, indicating the decomposition of organic materials derived from the cherry and pomegranate leaf extracts used in the synthesis. The inset in Figure 5b presents a comprehensive thermal profile for PP-FeHA, indicating its distinct exothermic behavior compared to other samples. All samples exhibited initial endothermic events below 200 °C, associated with dehydration, followed by notable thermal transitions at higher temperatures, indicating compositional differences and changes in thermal stability.

**Figure 6:**
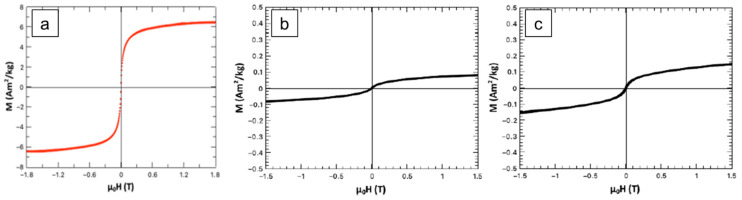
M-H curve/Hysteresis loop of (**a**) FeHA, (**b**) CP-FeHA, and (**c**) PP-FeHA.

Magnetic properties of the samples were assessed by AGFM (Alternating Gradient Force Magnetometry) (Figure 6). Magnetization curves confirm superparamagnetic behavior in all samples. FeHA (a) shows the highest magnetization (~7 Am^2^/kg), while CP-FeHA (b) and PP-FeHA (c) exhibit significantly reduced magnetization of ~0.10 and ~0.15 Am^2^/kg, respectively, as the iron oxide secondary phase is removed. The reduced magnetic response in the stabilized samples suggest that physicochemical interactions with plant extracts modulate magnetism through surface capping and/or altered Fe clustering.

We verified that the residual magnetization is still suitable for magnetic guiding, particularly if using magnetic catheters that can closely follow and direct the motion of FeHA nano-carriers.

It is interesting to note that pomegranate leaf extract allows the retention of slightly higher magnetization in the FeHA nanocarriers with respect to cherry leaf extract. The different behavior between the two extracts can be ascribed to specific interactions with Fe ions during FeHA nucleation and growth, likely affecting the interplay and dipolar interactions occurring at the crystal structure level that are responsible for the superparamagnetism in FeHA. Further studies in this respect can help to elucidate such interactions, relevant for supporting the design of magnetic nanocarriers with tailored magnetization extent.

### 2.3. Characterization of siRNA-Loaded PP-FeHA NPs and Release Properties

The isothermal adsorption of siRNA onto FeHA was carried out once the time to attain the thermodynamic adsorption equilibrium was determined. PP-FeHA NPs were chosen for siRNA functionalization because of their improved colloidal stability, higher antioxidant potential, and better magnetic properties, which collectively contribute to more efficient loading. In this experiment, the PP-FeHA-NPs/liquid ratio (1 mg, 1 mL) and temperature (37 °C) were kept the same as in the kinetic analysis, but the starting siRNA concentration was changed between 0.3 and 2.0 µM mL^−1^. The contact time was fixed at 120 min, then the NPs were separated from the supernatant by centrifugation.

**Figure 7:**
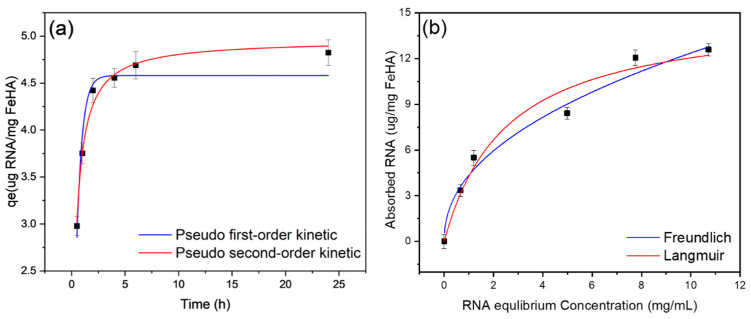
(**a**) Kinetic and (**b**) isotherm adsorption of siRNA on PP-FeHA-NPs.

The adsorption kinetics of siRNA on FeHA NPs is illustrated in Figure 7. The data shows a typical trend in which the loaded amount increases with time, first by a fast kinetic, followed by a gradual approach to equilibrium. At around 4.69 µg RNA/mg FeHA, a plateau is observed after 6 h, which indicates that the adsorption sites are saturated. The kinetic data have been adjusted to the commonly used models, namely the pseudo first-order and pseudo second-order models.

The fitted curves are shown in Figure 7a, and the corresponding parameters are described in Table 4. The adjusted R^2^ value of the pseudo second-order model (0.988) is much higher than that of the pseudo first-order model (0.956), indicating a better fit. This implies that the chemical interaction between adsorbent and adsorbate controls the loading process; the primary rate determining step is chemisorption [39].

These models are characterized as “pseudo” first/second-order kinetics since standard first/second-order kinetics have been proposed to explain the reactions of dissolved solutes in a liquid phase, but these models place emphasis on adsorbed species attached to a solid phase. The pseudo first-order model proposes that adsorption is reversible and that the rate of drug linked to the substrate over time is exactly proportional to the difference between the equilibrium quantity of drug adsorbed and the amount adsorbed at time t [40]. The mathematical equation for the pseudo first-order model is shown in Equation (1):

(1) $$Q\left(t\right)=Q_{ads,e}[1-e^{{-k}_{1}t}]$$

where *Q*(*t*) and *Q_ads,e_* represent the quantity of molecules adsorbed per unit weight of the sorbent (mg g^−1^) at time t and at equilibrium, respectively, whereas *k_1_* denotes the pseudo first-order adsorption rate coefficient (time^−1^).

The pseudo second-order kinetic model assumes that the rate-limiting phase is chemisorption, indicating that the adsorption rate depends on the presence of adsorption sites on the substrate rather than the concentration of the adsorbate and that the interaction is irreversible [40]. The mathematical formula for the pseudo second-order model is shown in Equation (2):

(2) $$Q\left(t\right)=\;Q_{ads,e}[1-\left(\frac{1}{1+k_{2}t}\right)]$$

In this case, *k_2_* represents the pseudo second-order adsorption rate coefficient (time^−1^).

Langmuir and Freundlich models were used to fit the isotherm data. A nonlinear least-squares fitting approach was used to derive the model parameters by fitting the curves to the experimental data. In the Langmuir adsorption model, certain assumptions are made, such as the following: (i) the adsorption capacity of solid particles is limited; (ii) every adsorption site is identical; (iii) every site maintains one molecule of the given substance; and (iv) every site is energetically and sterically independent from the adsorbed amount [41]. The mathematical representation of the Langmuir model is provided in Equation (3):

(3) $$Q=Q_{m}\frac{{(K}_{L}C_{e})}{1+\left(K_{L}C_{e}\right)}$$

where *Q* represents the number of molecules adsorbed per unit weight of the sorbent (mg g^−1^), *C_e_* is the equilibrium concentration of the adsorbate in solution (mg mL^−1^), *Q_m_* indicates the maximum loading capacity (mg g^−1^), and *K_L_* is the Langmuir affinity constant (mL mg^−1^).

The Freundlich model represents non-ideal and reversible adsorption, without the limitation of monolayer formation [41]. This empirical model is applicable to multilayer adsorption, characterized by a nonuniform distribution of adsorption sites and affinities over a heterogeneous surface. The Freundlich model is mathematically expressed in Equation (4):

(4) $$Q=K_{F}{C_{e}}^{1/n}$$

where *K_F_* represents the Freundlich affinity constant (mL mg^−1^), *C_e_* is the equilibrium concentration of the adsorbate in solution (mg mL^−1^), and *n* signifies the heterogeneity parameter.

The Korsmeyer–Peppas model is a semi-empirical equation used to characterize drug release kinetics from polymeric materials, especially when the release mechanism is complex or involves a combination of events. This concept applies to systems with diffusion-controlled or non-Fickian release patterns. The model is mathematically represented in Equation (5):

(5) $$M_{t}/M_{\infty }=Kt^{n}$$

where *M_t_*/*M_∞_* represents the proportion of drug released at time *t*, *K* denotes the release rate constant that covers the structural and geometric attributes of the delivery system, and n signifies the release exponent, which reflects the process of drug release. For spherical matrices, a *n* value ≤ 0.43 shows Fickian diffusion, 0.43 < *n* < 0.85 signifies anomalous (non-Fickian) transport, and *n* > 0.85 denotes case-II transport mostly influenced by polymer relaxation or swelling.

The adsorption isotherm curve for siRNA on FeHA, seen in Figure 7b, demonstrates a rapid initial loading of siRNA, followed by gradual stabilization up to about 12.06 µg of siRNA per mg of FeHA. Two well-known models, Freundlich and Langmuir, were used to fit the data, and the fitting parameters are reported in Table 5. The Langmuir model revealed an adjusted R^2^ of 0.963, a maximum loading capacity of 15 ± 1 µg RNA/mg FeHA, and a binding affinity constant of 0.4 ± 0.1 mL mg^−1^, indicating moderate binding affinity, a homogeneous surface, and monolayer coverage of siRNA. The data were further analyzed using the Freundlich model, which yielded a strong fit. The *n* value below 1 indicates negative cooperativity; therefore, when siRNA molecules adsorb onto the FeHA surface, they may partly prevent the attachment of more molecules, probably due to steric effects or charge repulsion. However, the Langmuir model presented a slightly better match; the n value from the Freundlich model demonstrates the surface heterogeneity of FeHA and the complexity of siRNA interaction kinetics. These findings indicate that surface irregularities and intermolecular effects affect the interaction, indicating a partially heterogeneous adsorption environment, even though siRNA linking to FeHA mostly follows a monolayer mechanism.

Based on the adsorption isotherm curves, a concentration of 12–15 µg RNA/mg FeHA and an incubation time of 6 h to reach near-equilibrium adsorption were selected for loading onto FeHA-NPs for further experiments.

siRNA was loaded to PP-FeHA NPs by adsorption method at two different incubation temperatures (37 °C and 42 °C) to investigate the influence of thermal conditions on formulation efficacy. siRNA-loaded PP-FeHA NPs incubated at 42 °C exhibited a slight increase in siRNA payload relative to those incubated at 37 °C, with a significant increase in siRNA loading efficiency, which could be due to the improved binding affinity to the PP-FeHA matrix at higher temperatures (Table 6). Stable and monodisperse siRNA–PP–FeHA NPs were developed in both conditions, showing negligible thermal effects on size, as evidenced by DLS and ζ-potential evaluation. Both formulations exhibited a net negative surface charge, with a slight increase in negativity at high temperatures. The ζ-potential of siRNA-PP-FeHA changed slightly from −30.3 ± 4.1 mV at 37 °C to −32.4 ± 3.3 mV at 42 °C, indicating a minor change in colloidal stability attributed to increased electrostatic repulsion among particles.

**Figure 8:**
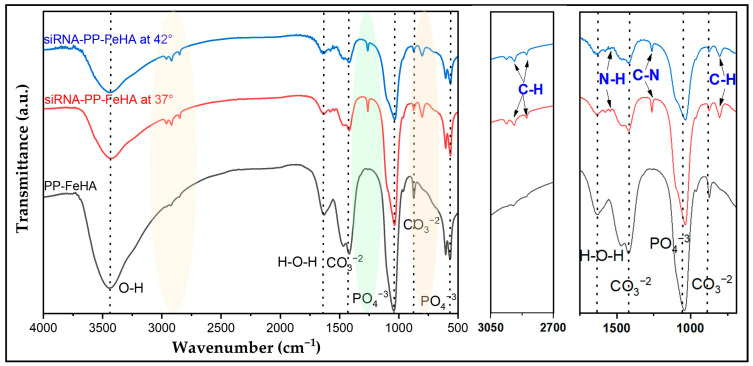
FTIR spectra of siRNA-PP-FeHA samples in comparison to PP-FeHA.

The FTIR spectrum of both siRNA-PP-FeHA samples in comparison to PP-FeHA (Figure 8) confirms that siRNA loading did not alter the apatitic structure of the materials nor the organic content. The presence of siRNA was confirmed by the presence of a distinct ~807 cm^−1^ peak in both siRNA-functionalized NPs, plausibly related to C–H out-of-plane bending [42], which was absent in PP-FeHA. Additional peaks appeared at ~2850–2950 cm^−1^, assigned to C–H stretching, and ~1264 cm^−1^, assigned to phosphate C-N stretching vibrations. Although the incorporation of siRNA without significant degradation was further confirmed by minor spectrum changes in the N-H region at 1540 cm^−1^, this method was unable to detect the adsorbed siRNA as its amount was below instrumental sensitivity [43]. 

**Figure 9:**
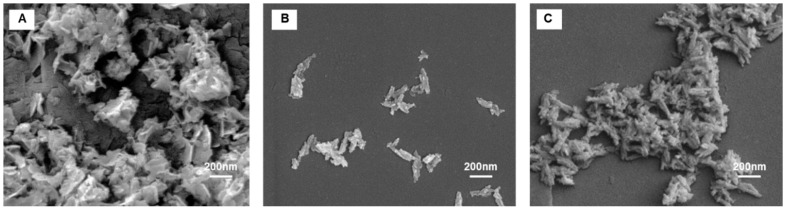
SEM images of (**A**) PP-FeHA, (**B**) siRNA-PP-FeHA-37 °C, and (**C**) siRNA-PP-FeHA-42 °C.

FE-SEM micrographs of siRNA-PP-FeHA (Figure 9) show a distinct morphology compared to PP-FeHA, with siRNA stabilizing particle interfaces and reducing aggregation. This suggests that siRNA not only acts as a therapeutic agent but also affects the NP shape and the surface charge, significantly reducing aggregation and promoting more defined surface features.

**Figure 10:**
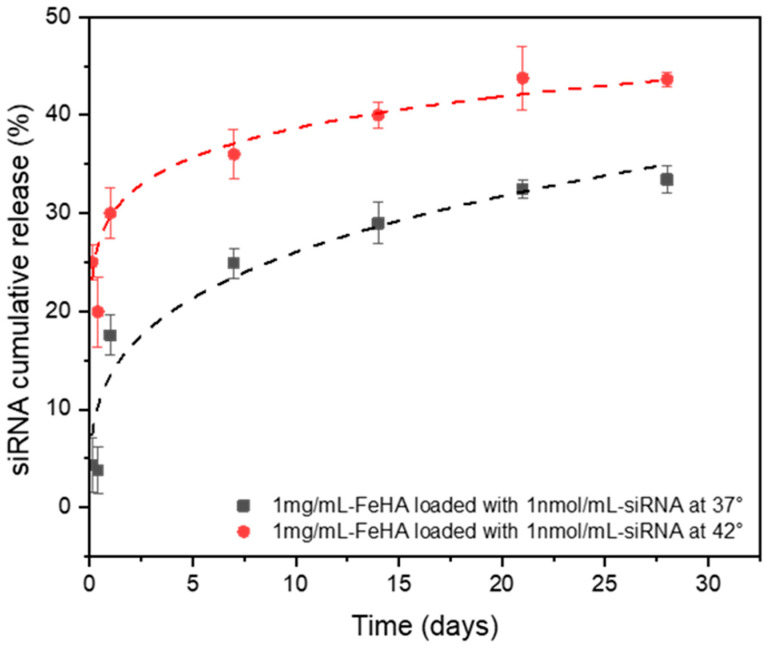
siRNA release from PP-FeHA NPs.

We evaluated the capacity of PP-FeHA to absorb and release siRNA over time by investigating its release kinetics in RNase-free water at two different temperatures: 37 °C (physiological) and 42 °C (mild hyperthermia) (Figure 10). Release curves collected over 4 weeks show an initial burst release of siRNA over 24 h due to a weakly absorbed siRNA on the NP surface, accounting for ca. 35% at 42 °C and about 17% at 37 °C of the total siRNA payload, indicating a temperature-dependent enhancement of release kinetics. This finding aligns with studies of thermally sensitive release mechanisms in nanoparticle drug delivery systems, whereby moderate hyperthermia enhances molecular diffusion and matrix swelling. Interestingly, following this initial phase, the two temperature conditions showed distinct trends. Over the 28-day duration, total siRNA release plateaued at around 44% at 42 °C by day 21, but the release at 37 °C was more gradual and sustained, reaching around 33%. The results suggest that moderate hyperthermia (42 °C) promotes faster siRNA desorption from PP-FeHA, a feature beneficial for on-demand release in hyperthermic therapeutic strategies [44]. The plateau observed at subsequent time intervals indicates a saturation point in the release profile at both temperatures. The temperature-dependent release characteristics of PP-FeHA emphasize its potential as a thermos-responsive delivery system for siRNA therapies, facilitating controlled release under physiological conditions and increased release under hyperthermic stimulation.

The siRNA release data were fitted to the Korsmeyer–Peppas model to evaluate the release mechanism (Table 7). The release patterns at both temperatures followed Fickian diffusion mechanisms closely, as shown by the release exponent (n) values being below 0.45 [45]. An increase in temperature led to a significantly higher release rate, indicated by a higher release constant and better model fit at 42 °C relative to 37 °C. The results indicate that siRNA release from the NP system is temperature-sensitive and mostly regulated by diffusion, with increased release under hyperthermic conditions. Although promising, these preliminary studies do not account for the challenges associated with siRNA stability in cell-relevant media, where exposure to nucleases would lead to rapid and irreversible degradation. Further investigations addressing biological performance and therapeutic efficacy will be essential for advancing this nanoplatform toward effective gene-delivery applications. In particular, the application of FeHA-based nanocarriers could be directed toward ex vivo cell therapy, where cells from a donor are treated with therapeutic nucleic acids in vitro and subsequently re-administered, allowing assessment of gene delivery efficacy without immediate systemic administration [46,47]. This approach will help bridge the gap between material characterization and potential gene therapy applications, highlighting the relevance of plant-extract functionalization for optimizing NP performance in therapeutic contexts.

## 3. Materials and Methods

### 3.1. Materials

Calcium hydroxide (Ca(OH)_2_, 96% pure), iron (II) chloride tetrahydrate (FeCl_2_·4H_2_O, ≥99.0% pure), iron (III) chloride hexahydrate (FeCl_3_·6H_2_O, ≥98.0% pure), orthophosphoric acid (H_3_PO_4_, ≥85% pure), o-phenanthroline (Merck 1,10-phenanthroline, ≥99% pure), and hydrochloric acid (HCl, 37%) were purchased from Sigma Aldrich (St. Louis, MO, USA). Model siRNA (Silencer™ GAPDH Positive Control siRNA, in vivo ready) and RNase-free water were purchased from Thermo Fisher Scientific (Waltham, MA, USA). All reagents were used without further purification.

Solutions were prepared with ultrapure water (18.2 MΩ × cm, 25 °C, Arium© pro, Sartorius, Gottingen, Germany).

### 3.2. Preparation of Cherry Plant (CP) and Pomegranate Plant (PP) Leaf Extracts

Fresh cherry and pomegranate leaves were first rinsed with water to remove any dirt and impurities. The leaves were then shade-dried at room temperature for a week before being finely pulverized. For the extraction, 10 g of each powdered leaf material was dissolved in 200 mL of distilled water, resulting in a powder-to-water ratio of 1:20. The mixture was stirred for an hour at 80 °C, and after that, the resulting mixture was filtered using 0.45-micron syringe filter to remove any solid particles. The clear leaf extract was then stored at 4 °C for further use in NP synthesis, where it served as both a stabilizing and reducing agent.

### 3.3. Synthesis of HA, FeHA, CP-FeHA, and PP-FeHA

The synthesis of iron-doped hydroxyapatite (FeHA), cherry plant-functionalized FeHA (CP-FeHA), and pomegranate plant-functionalized FeHA (PP-FeHA) NPs was carried out based on the methodology of Iannotti et al. [37], with modifications to incorporate plant extracts. Iron-free hydroxyapatite (HA) was synthesized as a control.

For synthesis of HA, an aqueous solution of H_3_PO_4_ (0.7 M, 75 mL) was added dropwise to an aqueous solution of Ca(OH)_2_ (0.79 M, 100 mL) under constant stirring and heating at 45 °C. The reaction continued for 3 h under heating and stirring, followed by overnight aging at room temperature. The resulting HA NPs were recovered by centrifugation (12,000 rpm for 5 min), washed four times with water, and freeze-dried.

For the synthesis of FeHA, the procedure was similar, but aqueous solutions of FeCl_2_∙4H_2_O (0.37 M, 20 mL) and FeCl_3_∙6H_2_O (0.37 M, 20 mL) were added simultaneously with the H_3_PO_4_ solution, maintaining the Fe/Ca molar ratio at 20 mol% and the Fe(III)/Fe(II) ratio at 1 to optimize the superparamagnetic ability of FeHA [16]. The pH was controlled at 9–10, and the reaction was heated and stirred for 3 h before aging overnight at room temperature. After centrifugation, washing, and freeze-drying, FeHA NPs were obtained.

**Figure 11:**
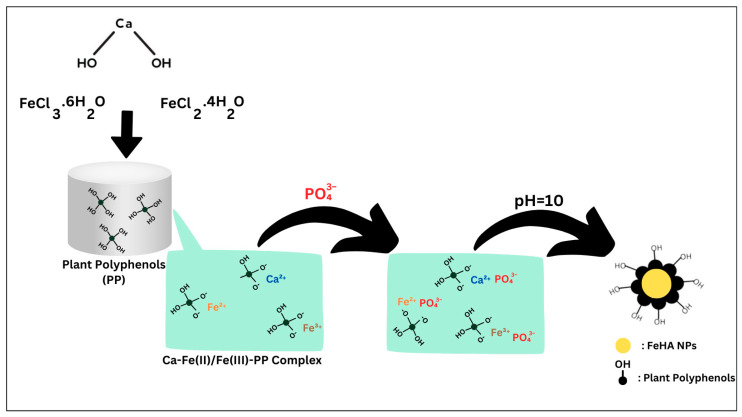
A schematic representation of the CP-FeHA and PP-FeHA preparation process.

For the synthesis of CP-FeHA and PP-FeHA, calcium (Ca(OH)_2_) and iron salts (FeCl_2_∙4H_2_O and FeCl_3_∙6H_2_O) in a 20 mol% Fe/Ca ratio were dissolved in the respective plant leaf extracts: cherry leaf extract (CLE) and pomegranate leaf extract (PLE). The plant extracts were used as solvents to stabilize the metal salts (Figure 11). The synthesis was carried out under the same conditions as FeHA, at 45 °C and pH 10, using the metal salt mixture dissolved in the plant extracts. The acid (H_3_PO_4_) was added dropwise at a rate of 3 mL/min using a peristaltic pump. The pH was checked using Whatman^®^ indicator papers and carefully controlled by adding a 1 M solution of sodium hydroxide (NaOH, Sigma Aldrich, ≥98.0% purity), as shown step by step in Figure 11. After the reaction, the NPs were recovered by centrifugation (12,000 rpm for 5 min), washed thoroughly with distilled water, and freeze-dried.

### 3.4. Determination of Antioxidant Activity Using the DPPH Assay

HPLC–UV Analysis. Quantitative analysis of the crude leaf extract phytochemicals was performed using High-Performance Liquid Chromatography (HPLC) on a 1260 Infinity II LC System (Agilent Technologies, Santa Clara, CA, USA), equipped with an Agilent Eclipse Plus C18 column (250 mm × 4.6 mm, 5 µm). The separation was achieved under isocratic conditions using a mobile phase consisting of 75% aqueous trifluoroacetic acid (0.065% *v*/*v*) and 25% acetonitrile. The flow rate was maintained at 1.0 mL·min^−1^, with the column temperature set at 25 °C. Detection was carried out at a wavelength of 254 nm (bandwidth 4 nm). A calibration curve was constructed using gallic acid as the reference standard and diluted 1:1 with 0.1 M HCl, reaching a concentration range of 5–100 ppm. The developed method demonstrated good linearity within this range. The retention time for the main gallic acid peak under these chromatographic conditions was approximately 2.9 min.

DPPH free radical scavenging assay. The antioxidant potentials of the pomegranate and cherry leaf extracts were evaluated using the DPPH (2,2-diphenyl-1-picrylhydrazyl) radical scavenging assay with some modifications [48]. A working DPPH solution was prepared at a concentration of 0.4 mM in 70% methanol (*v*/*v*). For the assay, 3 mL of the DPPH solution (0.4 mmol; in 70% methanol) was mixed and incubated with 200 μL of sample or standard solution for 30 min at room temperature. Then, the decrease in the absorbance compared to the control was monitored at 520 nm, using UV−visible spectrophotometry (Lambda 35 UV/vis spectrometer; PerkinElmer Instruments, Shelton, CT, USA).

Gallic acid was used as a standard antioxidant and a calibration curve was constructed using concentrations of 3, 6, 13, 19, 21, and 25 µg/mL in 70% methanol (*v*/*v*) by plotting absorbance against gallic acid concentration, and the resulting regression equation was used to calculate the antioxidant capacity. Extract concentrations were prepared at different concentration in 70% methanol (*v*/*v*) and treated under the same conditions as the standard.

DPPH radical scavenging activity of all prepared samples was calculated using the following equation:

$$DPPH\;radical\;scavenging\;activity\;(\%)=\frac{A_{C}-\;A_{S}}{A_{C}}\times 100$$

where *A_C_* is the absorbance of the control and *A_S_* is the absorbance of the sample.

The four-parameter logistic (4PL) regression model was used to determine the 50% inhibitory concentration (IC50) of free radical activity [49].

### 3.5. FeHA and PP-FeHA Characterization

X-Ray Powder Diffraction (XRPD). PXRD patterns of the dry samples were recorded on a D8 Advance diffractometer (Bruker, Karlsruhe, Germany) equipped with a Lynx-eye position sensitive detector in Bragg–Brentano geometry. Cu Kα radiation (*λ* = 1.54178 Å) generated at 40 kV and 40 mA was used. PXRD patterns were recorded in the 20–60° (2*θ*) angular range with a counting time of 0.5 s and a step size of 0.02°.

Fourier Transform IR Spectroscopy (FTIR). FTIR spectra of dry samples were collected in attenuated total reflectance (ATR) mode with a Nicolet iS5 spectrometer (Thermo Fisher Scientific Inc., Waltham, MA, USA) using an iD7 diamond ATR accessory. The spectra were collected with a resolution of 4 cm^−1^ by the accumulation of 32 scans covering the 4000 to 400 cm^−1^ spectral range. Splitting factors were calculated from FTIR spectra by dividing the sum of the absorbance of the peaks at 562 cm^−1^ and 602 cm^−1^ from ν_4_(PO_4_) bond bending by the absorbance of the minimum between these two peaks, using a similar method to the literature [35].

Field-Emission Scanning Electron Microscopy (FE-SEM). Micrographs of the samples were collected with a ZEISS ΣIGMA microscope (ZEISS NTS GmbH, Oberkochen, Germany) with InLens acquisition mode, operating at a 4 kV acceleration voltage, with a working distance of 3.5 mm and magnification between 100 Kx and 150 Kx. FeHA NPs were diluted with ultrapure water to a concentration of 0.1 mg mL^−1^. Afterward, a drop of NP suspension was deposited on a flat, mirror-polished silicon wafer mounted on an aluminum stub and dried at room temperature. Once the samples were dried, a ~10 nm gold layer was deposited by sputtering (Polaron E5100, Polaron Equipment, Watford, Hertfordshire, UK) under argon at 10^−3^ mbar for 1 min using a sputtering current of 30 mA, to provide electrical conductance.

Inductively coupled plasma optical emission spectrometry (ICP-OES). Quantification of Ca, P, and Fe was performed with an Agilent 5100 instrument (Agilent Technologies, Santa Clara, CA, USA). Before analysis, 10 mg of dry sample was dissolved in 50 mL of 2 wt.% HNO_3_ solution in triplicate.

UV-visible spectroscopy (UV–Vis). Quantification of Fe^2+^ content of the samples was performed through a colorimetric method involving o-phenanthroline (Merck 1,10-phenanthroline, ≥99% pure). Within a pH range of 4−5, ferrous ions react with o-phenanthroline to produce the stable red-orange complex [(C_12_H_8_N_2_)_3_Fe]^2+^, which can be detected at 510 nm using UV−visible spectrophotometry (Lambda 35 UV/vis spectrometer; PerkinElmer Instruments, USA). A 50 mg portion of FeHA powder was dissolved in 1 mL of 2 M HCl (ACS reagent, 37%) after confirming that hydrochloric acid did not influence the concentration of the Fe^2+^-complexed molecule, at least for the period of the experiment. A 10 mL portion of sodium citrate buffer (0.1 M, pH 4) was added into the hydrochloric acid solution to maintain the pH at approximately 4–5 and to inhibit the oxidation of Fe^2+^; thereafter, a suitable volume of 0.2 wt.% o-phenanthroline solution was added to achieve a nominal Fe^2+^/o-phenanthroline molar ratio of 1/3. The total volume of the final solution increased to 50 mL by adding ultrapure water. The difference between the overall quantity of Fe (measured by ICP) and the quantity of Fe^2+^ (measured by UV–Vis) was used to determine the amount of Fe^3+^.

Thermogravimetric analysis (TGA). Analyses were performed using STA 449C Jupiter (Netzsch GmbH, Selb, Germany) apparatus. Ca. 10 mg of dry sample was weighed in an alumina crucible and then heated from room temperature to 1100 °C under air flow with a heating rate of 10 °C/min.

Specific Surface Area (SSA_BET_). SSA_BET_ was measured through N_2_ gas adsorption method using a Surfer instrument (Thermo Fisher Scientific Inc., Waltham, MA, USA) and Brunauer–Emmett–Teller method. Before measurement, samples were degassed at 200 °C for 3 h under vacuum.

Magnetic characterization. The samples were characterized by measuring hysteresis loops by Alternating Gradient Force Magnetometry (AGFM) at room temperature. For each analysis, ultra-thin plastic substrates of about 3 × 3 mm^2^ were used; a small amount of powder (0.20–0.40 mg) was deposited on these, which was then fixed to the substrate with a small amount of cyanoacrylate. The magnetization curve was measured for each sample in hysteresis loop mode (from +μ_0_H_max_ to −μ_0_H_max_ and then from −μ_0_H_max_ to +μ_0_H_max_) in a maximum magnetic field μ_0_H_max_ equal to 2 T and with a field variation step equal to 100 Oe. To obtain the final graphs, the following were subtracted from the measured magnetic moment: the diamagnetic contribution of the measuring probe, that of the plastic substrate, and that of the cyanoacrylate. The subtraction occurred after having appropriately re-proportioned the last two contributions with respect to the sample weight, considering the relative weights of the sample, substrate, and cyanoacrylate in each prepared sample. Starting from the measured magnetic moment, the magnetization of the sample in Am^2^/kg was obtained, using the weight of the sample. The measuring probe (parallel probe) was calibrated at the beginning of each measurement day with a known time standard (ultrapure Pt disk).

Dynamic Light Scattering (DLS) and electrophoretic mobility. The measurement of NPs’ hydrodynamic diameter distribution and electrophoretic mobility (ζ-potential) was performed using a Zetasizer Nano ZSP instrument (Malvern Instruments, Malvern, UK). Samples were analyzed in suspension at 1 mg mL^−1^ concentration and at pH 7. The hydrodynamic diameter distribution of the samples at 25 °C was measured using hydroxyapatite and water refractive indexes (1.63 and 1.33) as working parameters for the samples and the solvent, respectively, for three measurements of at least 10 runs. Particle size is reported as Z-average of hydrodynamic diameter distribution. ζ-potentials were quantified as the electrophoretic mobility at 25 °C of three separate measurements (maximum 100 runs each) by laser Doppler velocimetry using a disposable electrophoretic cell (DTS1061, Malvern Ltd., Worcestershire, UK) with the same sample and solvent parameters.

### 3.6. siRNA Loading on PP-FeHA

Prior to adsorption, freeze-dried siRNA was dissolved in RNase-free water to prepare a 100 µM stock solution. The PP-FeHA suspension was diluted to 1 mg/mL in a total volume of 5 mL, and 50 µL of siRNA was added to achieve a final concentration of 1 µM (approximately 13 µg/mg of PP-FeHA). The suspension was then incubated for 1 h at both 37 °C and 42 °C to form siRNA-PP-FeHA NPs. To remove non-adsorbed siRNA, the suspension was centrifuged at 5000 rpm for 2 min; the supernatant was discarded, and the pellet was resuspended in RNase-free water. The resulting NPs were subsequently stored at −20 °C for further use.

### 3.7. Characterization of siRNA-PP-FeHA and siRNA Release

siRNA quantification. Adsorbed siRNA was measured indirectly by quantification of the non-adsorbed fraction in the supernatant. siRNA quantification was performed by using the Quant-it microRNA assay kit (Thermo Fisher Scientific Inc., Waltham, MA, USA) according to the manufacturer’s instructions. A calibration curve of siRNA between 0 and 2 µM was used. Fluorescence intensity was measured with a Fluoroskan Microplate Fluorometer (Thermo Fisher Scientific Inc., Waltham, MA, USA) at 485/538 nm excitation/emission wavelengths. Three replicates were performed for each sample. Adsorbed siRNA is expressed both as adsorption efficiency, i.e., weight percentage relative to used siRNA mass, and as payload, i.e., weight percentage (wt.%) relative to the mass of PP-FeHA NPs.

siRNA release from siRNA-PP-FeHA NPs. In total, 5 mg of siRNA-PP-FeHA were dispersed into 5 mL of RNase-free water at a final concentration of 1 mg/mL, respectively, in triplicate. The suspension was maintained at 37 °C and 42 °C under horizontal shaking. At scheduled times (5 h, 1 day, and 1, 2, 3, and 4 weeks) NPs were separated from the liquid phase by centrifugation (5000 rpm, 2 min) and 200 µL of the supernatant was removed for siRNA quantification as reported above. After that, 200 µL of fresh RNase-free water was added and the pellet was resuspended by vortexing gently at low speed, and the suspension was again put under agitation until the subsequent time point.

### 3.8. Study of siRNA Loading on FeHA-NPs

The adsorption study was conducted in two phases: first, to examine the kinetics of adsorption to determine the time required to achieve thermodynamic adsorption equilibrium, and subsequently, to analyze the adsorption isotherms. An adsorption kinetic study of siRNA on PP-FeHA was carried out by mixing 1 mL of 1 µM mL^−1^ siRNA solution and 1 mL of 1 mg mL^−1^ FeHA suspension. Samples were maintained at 37 °C under horizontal shaking. At scheduled times ranging from 30 min to 24 h, NPs were separated from the liquid phase by centrifugation (5000 rpm, 2 min) and 200 µL of the supernatant was removed for siRNA quantification.

The kinetic data was analyzed using standard models of pseudo first-order and pseudo second-order kinetics. The curves were adjusted to the experimental data via a nonlinear least-squares fitting approach to obtain the values for the model parameters.

## 4. Conclusions

The present paper deals with the efficiency of natural extracts (namely pomegranate and cherry leaves) able to modulate the nucleation and crystallization pathway of hydroxyapatite NPs, resulting in improved colloidal stability of CaP NPs, relevant for functioning as nanocarriers for nucleic acids. Specific focus was placed on using Fe(II)/Fe(III)-doped apatite (FeHA) which, thanks to its intrinsic superparamagnetism, could enable magnetic guiding to specific target organs/tissues.

In this respect, we found marked antioxidant properties in both the plant extracts, which are very important for therapeutic purposes but also for the modulation of the colloidal and magnetic properties of FeHA nanocarriers. Pomegranate leaf extracts in particular show the ability to limit the oxidation extent of Fe(II) to Fe(III) during the incorporation of iron ions into the calcium phosphate structure. This phenomenon triggers higher magnetization of the siRNA-PP-FeHA complex, which is promising for in vivo magnetic guiding.

In addition, the functionalization with plant extracts yielded enhanced surface charge, improving the colloidal stability and reducing particle agglomeration. Such a characteristic is very important for in vivo application while assuring at the same time a high level of cytocompatibility.

Our results show that plant extracts are promising alternatives to the more widely used citrate molecules for the preparation and colloidal stabilization of delivery systems made of inorganic nanoparticles.

Increasing the surface charge and specific surface area of FeHA, pomegranate leaf extract in particular permitted a higher extent of siRNA loading and the ability of sustained release along several weeks.

In addition, we found that the release extent of the siRNA can be significantly enhanced by a slight temperature rise, thus revealing a thermo-responsive behavior that makes FeHA–plant nanocarriers very promising for spontaneously responding to pathological conditions, such as inflammation and tumors, or in the case of mild hyperthermia therapies. In conclusion, FeHA NPs functionalized with plant extracts show interesting perspectives for applications in nanomedicine, and extensive biological validation will be necessary to substantiate this potential. In this study, we proved the significant potential of plant extract-stabilized FeHA NPs as gene delivery nanomaterials, presenting a promising theranostic platform that integrates bioinspired materials, genetic nanomedicine, and magnetic guiding. Further studies will focus on comprehensive in vitro characterization, including cellular uptake and transfection efficiency, to further evaluate its viability as a magnetically guided siRNA delivery system. Concurrently, the impact of magnetic field parameters, including intensity, type (pulsed, alternating, static), frequency, and extent of magnetization, should be assessed to further elucidate the therapeutic potential of the nanoplatform. The progress of these studies will also contribute to advances in the growing research field on magnetic biomaterials.

## Figures and Tables

**Table 1:** DPPH IC50 value and four-parameter logistic model parameters for antioxidants.

Antioxidants	DPPH IC50 (µg/mL)	Adjusted R^2^
Gallic acid	54.23 ± 4	0.991
Pomegranate leaf extract	145.84 ± 10	0.988
Cherry leaf extract	349.3 ± 30	0.993

**Table 2:** ICP-OES and UV–Vis analysis of the synthesized samples.

Sample	Ca/P (mol) ^a^	(Ca + Fe)/P (mol) ^a^	Fe (wt.%) ^a^ *	Fe^2+^ (wt.%) ^b^ *	Fe^3+/^Fe^2+^	Fe^2+/^Fe^3+^	Fe^2+^ % (Fe^2+^/Fe tot)	Fe^3+^ % (Fe^3+^/Fe tot)
**HA**	1.69 ± 0.01	1.69 ± 0.01	-	-	-	-	-	-
**FeHA**	1.47 ± 0.01	1.78 ± 0.02	13 ± 1	0.74 ± 0.01	16.57	0.06	5.69	94.29
**CP-FeHA**	1.64 ± 0.01	1.81 ± 0.01	5 ± 0.1	0.37 ± 0.01	12.51	0.08	7.40	92.60
**PP-FeHA**	1.63 ± 0.01	1.90 ± 0.03	7.8 ± 0.4	0.96 ± 0.02	7.12	0.14	12.31	87.69

^a^ From ICP-OES analysis. ^b^ From UV–Vis analysis. * Fe wt% for CP/PP-FeHA normalized by TGA-based organic content.

**Table 3:** Mass loss (in weight % ± 0.01) obtained from TGA, BET surface area, hydrodynamic diameter, and surface charge of the synthesized samples.

Sample	SSA_BET_ (m^2^/g)	Z-Average (nm) ^b^	PdI ^b^	ζ-Potential (mV) ^b^	Water Loss % (20–250 °C) ^a^	Organic Decomposition % (250–500 °C) ^a^	Carbonate Loss % (600–1100 °C) ^a^	Total Loss % (20–1100 °C) ^a^
**HA**	97.18	220 ± 1	0.27 ± 0.01	−18 ± 3	5.14	N/A	1.98	7.11
**FeHA**	102.65	195 ± 1	0.26 ± 0.05	−24 ± 3	4.96	N/A	2.58	7.54
**CP-FeHA**	182	80 ± 1	0.29 ± 0.02	−32 ± 2	8.21	4.9	3.20	16.27
**PP-FeHA**	235.75	88 ± 1	0.18 ± 0.01	−35 ± 4	7.75	6.4	7.61	21.75

^a^ From TGA. ^b^ From DLS analysis.

**Table 4:** Adsorption isotherm parameters calculated from the curve fitting of experimental data according to the pseudo first-order and pseudo second-order models.

Model	Adjusted R^2^	Q_ads,e_ (mg RNA/g FeHA)	k1/k2 (min^−1^)
**Pseudo first-order**	0.956	4.58 ± 0.09	1.96 ± 0.15
**Pseudo second-order**	0.988	4.96 ± 0.06	0.62 ± 0.05

**Table 5:** Adsorption isotherm parameters calculated from the curve fitting of experimental data according to the Langmuir and Freundlich models.

Model	Adjusted R^2^	Q_m_ (µg RNA/mg FeHA)	KL/KF (µM^−1^)	n
**Langmuir**	0.963	15.09 ± 1.61	0.40 ± 0.13	-
**Freundlich**	0.959	-	4.51 ± 0.53	0.44 ± 0.058

**Table 6:** Adsorbed siRNA content and colloidal suspension parameters of siRNA-PP-FeHA samples.

Sample	siRNA Payload (nmol/mg)	siRNA Loading Efficiency (%)	Z-Average (nm)	PdI	ζ-Potential (mV)
siRNA-PP-FeHA at 37°C	0.57 ± 0.04	75.00 ± 0.32	179.0 ± 1.2	0.22 ± 0.01	−30.3 ± 4.1
siRNA-PP-FeHA at 42°C	0.63 ± 0.03	88.00 ± 0.20	180.0 ± 1.5	0.26 ± 0.02	−32.4 ± 3.3

**Table 7:** Korsmeyer–Peppas model parameters for siRNA release from FeHA.

Sample	K (Release Constant)	*n* (Release Exponent)	Release Mechanism	R-Square (COD)
**siRNA-PP-FeHA at 37°**	0.135 ± 0.020	0.286 ± 0.050	Fickian Diffusion	0.93
**siRNA-PP-FeHA at 42°**	0.296 ± 0.011	0.116 ± 0.012	Fickian Diffusion	0.96

## Data Availability

The original contributions presented in this study are included in the article. Further inquiries can be directed to the corresponding author.
